# Targeting proteasome-associated deubiquitinases as a novel strategy for the treatment of estrogen receptor-positive breast cancer

**DOI:** 10.1038/s41389-018-0086-y

**Published:** 2018-09-24

**Authors:** Xiaohong Xia, Yuning Liao, Zhiqiang Guo, Yanling Li, Lili Jiang, Fangcheng Zhang, Chuyi Huang, Yuan Liu, Xuejun Wang, Ningning Liu, Jinbao Liu, Hongbiao Huang

**Affiliations:** 1 Guangzhou Institute of Cardiovascular Disease, The Second Affiliated Hospital of Guangzhou Medical University, Guangzhou, Guangdong 510260 China; 2 Key Laboratory of Protein Modification and Degradation of Guangdong Higher Education Institutes, School of Basic Medical Sciences; Guangzhou Institute of Oncology, Tumor Hospital, Guangzhou Medical University, Guangzhou, Guangdong 511436 China; 3 Division of Basic Biomedical Sciences, Univeristy of South Dakota Sanford School of Medicine, Vermillion, SD 57069 USA

## Abstract

Estrogen receptor α (ERα) is expressed in ~67% of breast cancers and is critical to their proliferation and progression. The expression of ERα is regarded as a major prognostic marker, making it a meaningful target to treat breast cancer (BCa). However, hormone receptor-positive BCa was sometimes irresponsive or even resistant to classic anti-hormonal therapies (e.g., fulvestrant and tamoxifen). Hence, novel anti-endocrine therapies are urgent for ERα^+^ BCa. A phase II study suggested that bortezomib, an inhibitor blocking the activity of 20 S proteasomes, intervenes in cancer progression for anti-endocrine therapy in BCa. Here we report that proteasome-associated deubiquitinases (USP14 and UCHL5) inhibitors b-AP15 and platinum pyrithione (PtPT) induce growth inhibition in ERα^+^ BCa cells. Further studies show that these inhibitors induce cell cycle arrest and apoptosis associated with caspase activation, endoplasmic reticulum (ER) stress and the downregulation of ERα. Moreover, we suggest that b-AP15 and PtPT block ERα signaling via enhancing the ubiquitin-mediated degradation of ERα and inhibiting the transcription of ERα. Collectively, these findings demonstrate that proteasome-associated deubiquitinases inhibitors b-AP15 and PtPT may have the potential to treat BCa resistant to anti-hormonal therapy.

## Introduction

Breast cancer (BCa) is a common malignancy and the second most deadly cancer among women, with an increasing incidence worldwide^1,2^. It is well known that roughly 70% of BCa cells express estrogen receptor alpha (ERα) and sex hormones, which are critical to breast carcinogenesis^3–5^. While overall five-year survival rates for breast cancer have increased by almost 20% since 1975, largely because of the development of endocrine therapy^6^. For example, tamoxifen and trastuzumab are effective chemicals against estrogen receptor and Her2/Neu receptor, respectively. In addition, a lot of compounds were studied on ER-positive (ER^+^) breast cancer cells^7–10^. Despite of these recent advances, some of patients did not respond to the treatment. Therefore, identifying alternative strategies is a current challenge and an urgent need for the treatment of BCa.

Ubiquitin-mediated degradation is a major manner of protein degradation that strictly controls the quality and quantity of cellular proteins. Generally, proteins are selectively labeled by ubiquitin (polypeptide) and the ubiquitinated proteins are then recognized and degraded by the proteasome. The ubiquitin proteasome system consists of ubiquitin-conjugating complexes, deubiquitinating enzymes (DUBs) and the proteasome. Proteasome inhibitors have been successfully used to treat cancer in the clinic. For example, bortezomib has become a brilliant drug against multiple myeloma. DUBs hydrolyze ubiquitin chains and prevent protein degradation by deubiquitinating protein substrates. DUBs are involved in multiple physiological and pathological processes via regulating substrates of signal transduction. Recently, a series of small molecules against DUBs have been developed and implicated in cancer treatment^11^. In mammalian cells, three deubiquitinating enzymes (USP14, UCHL5, RPN11) associate with the 19 S proteasome. RPN11 is regarded as an intrinsic subunit of the 19S proteasome. USP14 and UCHL5 are reversibly recruited and activated (as DUBs) by the 19 S proteasome, which were identified as novel targets for anti-cancer.

Platinum pyrithione (PtPT) and b-AP15 have been defined as 19 S DUBs inhibitors, targeting USP14 and UCHL5^12,13^ without effect on the 20 S proteasome. Both b-AP15 and PtPT exert potent anti-cancer effects within a dose range that is biologically safe^13–15^. Our previous study also showed the anti-tumor potential of PtPT on Bcr–Abl-positive cell lines^16^. In the current study, we provide a potential strategy to anti-ERα^+^ BCa by the use of two inhibitors of proteasome-associated DUBs.

## Results

### USP14 and UCHL5 inhibitors suppress the growth of ER^+^ BCa cells

**Fig. 1:**
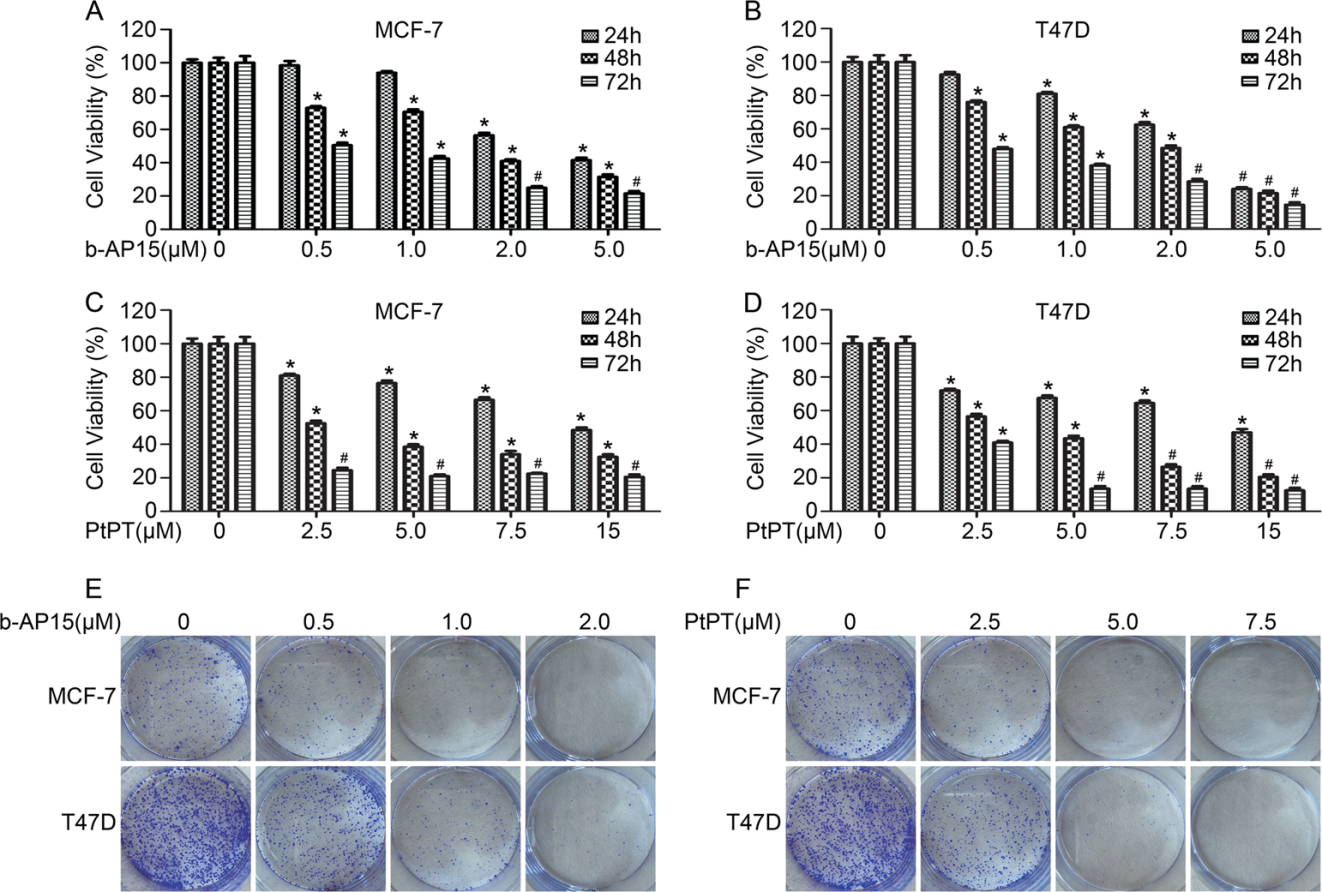
b-AP15 and PtPT suppress the growth of BCa cells. **a**, **b** BCa cells were treated with or without b-AP15. **c**, **d** BCa cells were treated with or without PtPT. Cell viability was tested using MTS assays. Mean ± SD of three independent experiments. *p < 0.05, ^#^*p* < 0.01, the two-sided t-test. BCa cells were treated with b-AP15 (**e**) and PtPT (**f**) for 24 h, then were transplanted in 30% agarose for 10 days. The shown images were from one of three independent experiments

Proteasome associated deubiquitinases have emerged as novel targets for cancer treatment. To evaluate the effects of USP14 and UCHL5 inhibitors on ER^+^ human BCa cells. We firstly detected the cell viability of BCa cells exposed to b-AP15 and PtPT, two selective inhibitors of USP14 and UCHL5 reported recently, using MTS assay. Notably, Both b-AP15 (0.5, 1, 2, 5 μM) and PtPT (2.5, 5, 7.5, 15 μM) suppressed the cell viability of ER^+^ MCF-7 and T47D cells (Fig. 1a–d). The effect of b-AP15 and PtPT in triple negative breast cancer (TNBC) and ER^−^/HER2^+^ breast cancer were detected through the above assay. We found that the loss of cell viability of MDA-MB468 and MDA-MB453 cells were induced (Supplementary Fig. S1a–d). To further detect the ability of colony formation of ER^+^ BCa cells upon inhibition of USP14 and UCHL5, we observed the growth of MCF-7 and T47D colonies for 10 days after b-AP15 and PtPT treatment for 24 h. As increasing concentration of b-AP15 and PtPT, the colonies were notably decreased (Fig. 1e, f). In addition, ER^+^ BCa cells exposed to b-AP15 and PtPT were treated with estrogen. MTS assay showed that b-AP15/ PtPT decreases the response to estrogen in MCF7 and T47D cells (Supplementary Fig. S2a, b). Studies have been reported that tamoxifen is the only guideline in endocrine therapy via ERα inhibiton. To further explore the roles of b-AP15 and PtPT in ER^+^ BCa cells, we performed the combination treatment (b-AP15 or PtPT + tamoxifen). The results showed that the cell viability in combined treatment group was lower than in separate treatment group (Supplementary Fig. S2c, d). Moreover, we detected the effect of bortezomib in treated cells by b-AP15 or PtPT. We found that b-AP15 or PtPT did not significantly increase the sensitivity of bortezomib in MCF7 and T47D cells (Supplementary Fig. S2e, f). Our results suggest that cell growth was dose- and time- dependently suppressed by b-AP15 and PtPT in ER^+^ BCa cells. What was significant was that USP14 and UCHL5 inhibitors enhanced the sensitivity of tamoxifen in MCF-7 and T47D cells.

### Inhibition of USP14 and UCHL5 activates caspase and triggers apoptosis of ER^+^ BCa cells

**Fig. 2:**
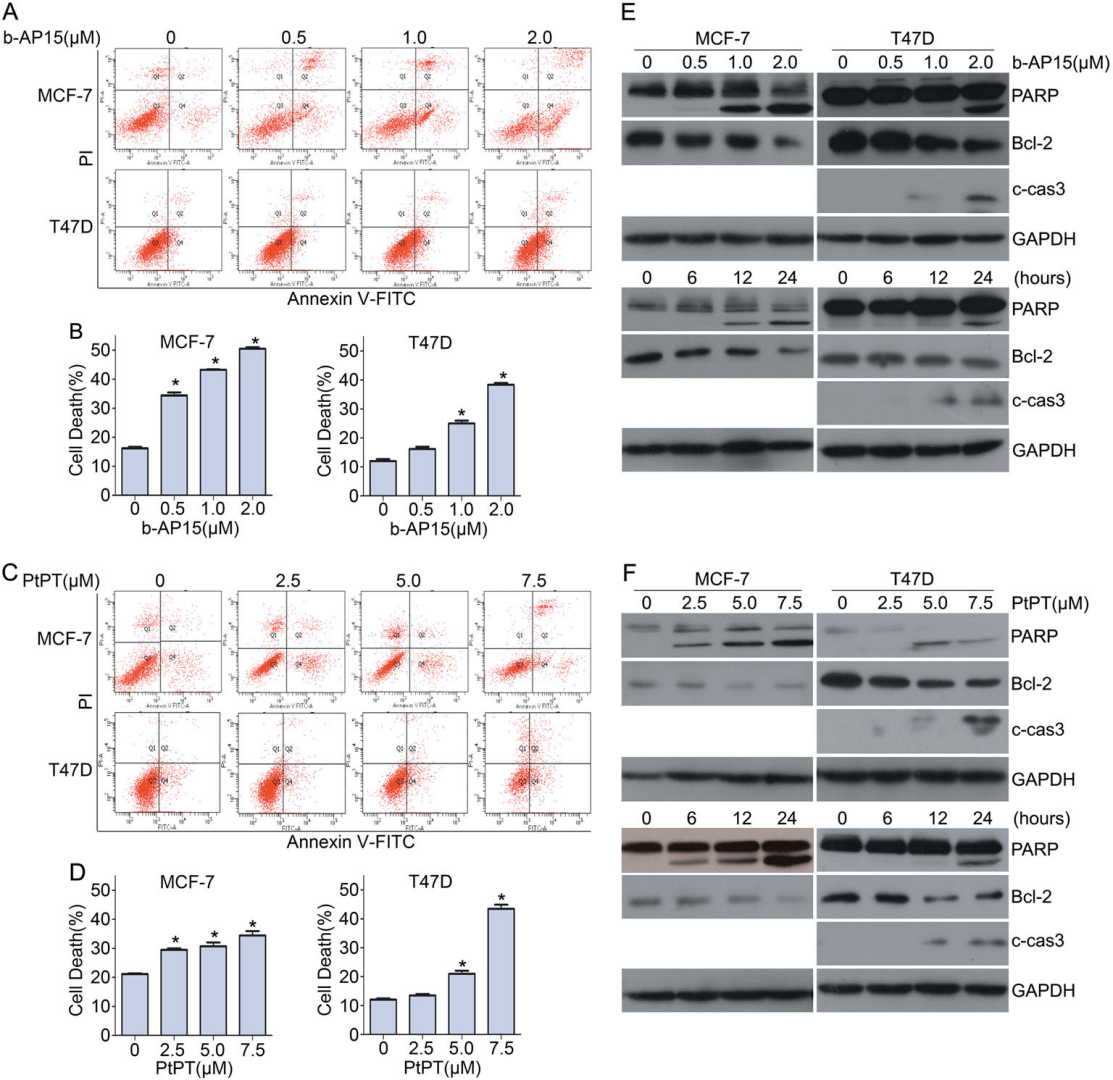
Inhibition of USP14 and UCHL5 induces cell death in BCa cells. **a** BCa cells were treated with the indicated concentrations of b-AP15 for 24 h. Cells were harvested and stained with Annexin V-FITC/PI followed by flow cytometry. Representative images and **b** quantification of cell death were shown. Mean ± SD of three independent experiments. *p < 0.05. **c** BCa cells were exposed to the indicated concentrations of PtPT for 24 h. Cells were collected and stained with Annexin V-FITC/PI followed by flow cytometry. Representative images and (**d**) quantification of cell death were shown. Mean ± SD of three independent experiments. *p < 0.05. **e** BCa cells were treated with indicated concentrations of b-AP15, or b-AP15 (1 μM) for the indicated time. Total proteins were extracted. The expression of PARP, Bcl-2 and cleaved caspase-3 were tested by western blot. GAPDH was used as a loading control (**f**) BCa cells were treated with indicated concentrations of PtPT, or PtPT (5 μM) for indicated time. Total proteins were extracted. The expression of PARP, Bcl-2 and cleaved caspase-3 were tested by western blot. GAPDH was shown as a loading control

Given that b-AP15 and PtPT inhibit cell viability of ER^+^ human BCa cells, identifying the underlying pathways involved is critical. We further asked whether b-AP15 and PtPT induce cell death of ER^+^ BCa. Using flow cytometry analysis under Annexin-V FITC/propidium iodide (PI) staining, we tested the effect of b-AP15 and PtPT on apoptosis induction in MCF-7 and T47D. Both b-AP15 and PtPT significantly induced apoptosis of these cells (Fig. 2a–d). To investigate whether the induction of apoptosis was associated with caspase activation, we detected the expression of activated caspase 3, Bcl-2 and PARP in MCF-7 and T47D cells upon b-AP15 and PtPT treatment using western blotting(MCF7 is deficient of caspase 3). We found that b-AP15 and PtPT dose- and time- dependently induced caspase-3 activation, PARP cleavage and BCl-2 downregulation (Fig. 2e, f).These results indicate that b-AP15 and PtPT trigger apoptosis in ER^+^ BCa cells and this is associated with caspase activation and mitochondria malfunction.

### USP14 and UCHL5 inhibitors induce accumulation of ubiquitinated proteins and endoplasmic reticulum stress response in ER^+^ BCa

**Fig. 3:**
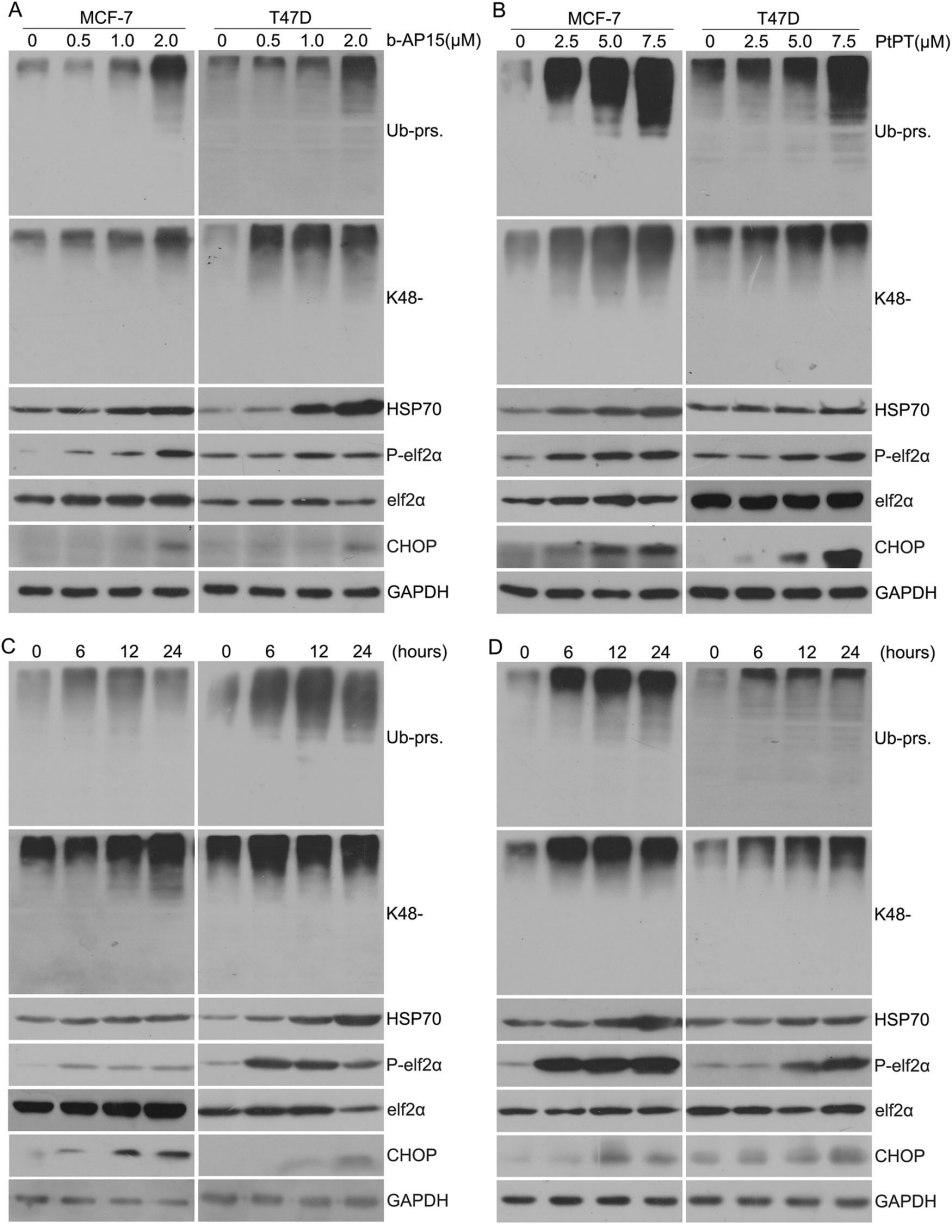
b-AP15 or PtPT trigger ER stress in BCa cells. **a**, **b** BCa cells were treated with b-AP15 (0, 0.5, 1, 2 μM) or PtPT (0, 2.5, 5, 7.5 μM) for 24 h. Proteins lysates were subjected to western blot analysis for total ubiquitinated proteins (Ub-prs), K48-linked ubiquitin, P-eIF2α, eIF2α, HSP70,CHOP. GAPDH was shown as a loading control. **c**, **d** BCa cells were treated with b-AP15 (1 μM) or PtPT (5 μM) for 0, 6, 12, 24 h. The expression of proteins as mentioned above was detected using western blot analysis

Previously, we showed that b-AP15 and PtPT are potent and selective inhibitors of UCHL5 and USP14^12,13^. The current study has further investigated the ability of b-AP15 and PtPT on proteasome inhibition and unfolded proteins response in ER^+^ BCa. We tested the accumulation of ubiquitinated proteins (Ub-prs) on ER^+^ BCa cells treated with b-AP15 and PtPT using western blot. Notably, both b-AP15 and PtPT accumulate pan- and K48-linked ubiquitin conjugates. Meanwhile, endoplasmic reticulum (ER) stress response was triggered by b-AP15 and PtPT. Our experiments results show that b-AP15 and PtPT treatment gave rise to changes in several proteins that are indicative of ER stress, including increases of C/EBP homology protein (CHOP) and phosphorylated eIf2α (P-eIf2α) accompanied by an increase in HSP70, which mediates cell homeostasis when cells are undergoing stress (Fig. 3). The findings indicate that cell apoptosis induced by b-AP15 and PtPT was associated with not only caspase activation but also ER stress.

### USP14 and UCHL5 inhibitors suppress cell cycle progression

**Fig. 4:**
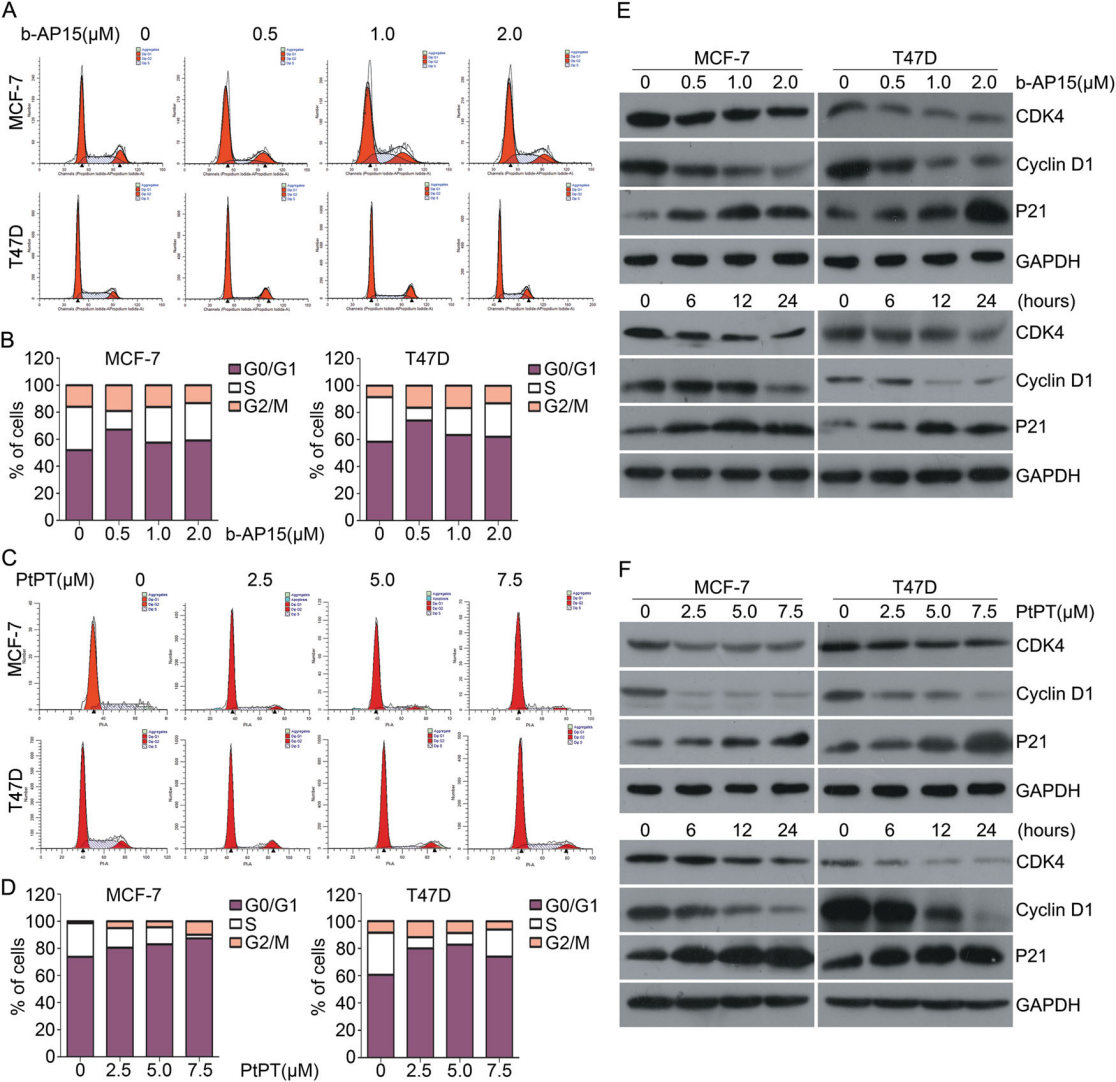
b-AP15 or PtPT induce cell cycle arrest in BCa cells. **a** BCa cells were exposed to b-AP15 for 24 h. Cells were fixed and stained with PI followed by flow cytometry. Representative images and (**b**) distribution of cell cycle stages is shown. **c** BCa cells were exposed to PtPT for 24 h. Cells were fixed and stained with PI followed by flow cytometry. Representative images and (**d**) distribution of cell death are shown. **e** BCa cells were treated with indicated concentrations of b-AP15, or b-AP15 (1 μM) for indicated time. Total proteins were extracted. The expression of CDK4, cyclinD1 and P21 were tested by western blot. GAPDH was shown as a loading control. **f** BCa cells were treated with indicated concentrations of PtPT, or PtPT (5 μM) for the indicated time. Total proteins were extracted. The expression of CDK4, cyclinD1 and P21 were tested by western blot. GAPDH was probed as a loading control

To further understand the mechanisms of cells growth inhibition induced by b-AP15 and PtPT, flow cytometry was used to test cell cycle progression. ER^+^ BCa cells were treated with various concentrations of the inhibitor for 24 h and harvested for for cell cycle analysis. Remarkably, b-AP15 and PtPT arrest cell cycle at G0/G1 phase (Fig.4a–d). Subsequently, we tested the dose-response and the time course of the effect of b-AP15 and PtPT treatment on the proteins related to the G1–S phase transition. Western blot analyses confirmed that b-AP15 and PtPT dose- and time-dependently downregulated the protein levels of CDK4 and cyclin D1 but increased the expression of p21. The above results indicated that b-AP15 and PtPT inhibit G1–S phase transition in MCF-7 and T47D cells through downregulating CDK4 and cyclin D1 and upregulating p21 expression (Fig. 4e, f).

### USP14 and UCHL5 inhibitors downregulate ERα and IGF-1R expression

**Fig. 5:**
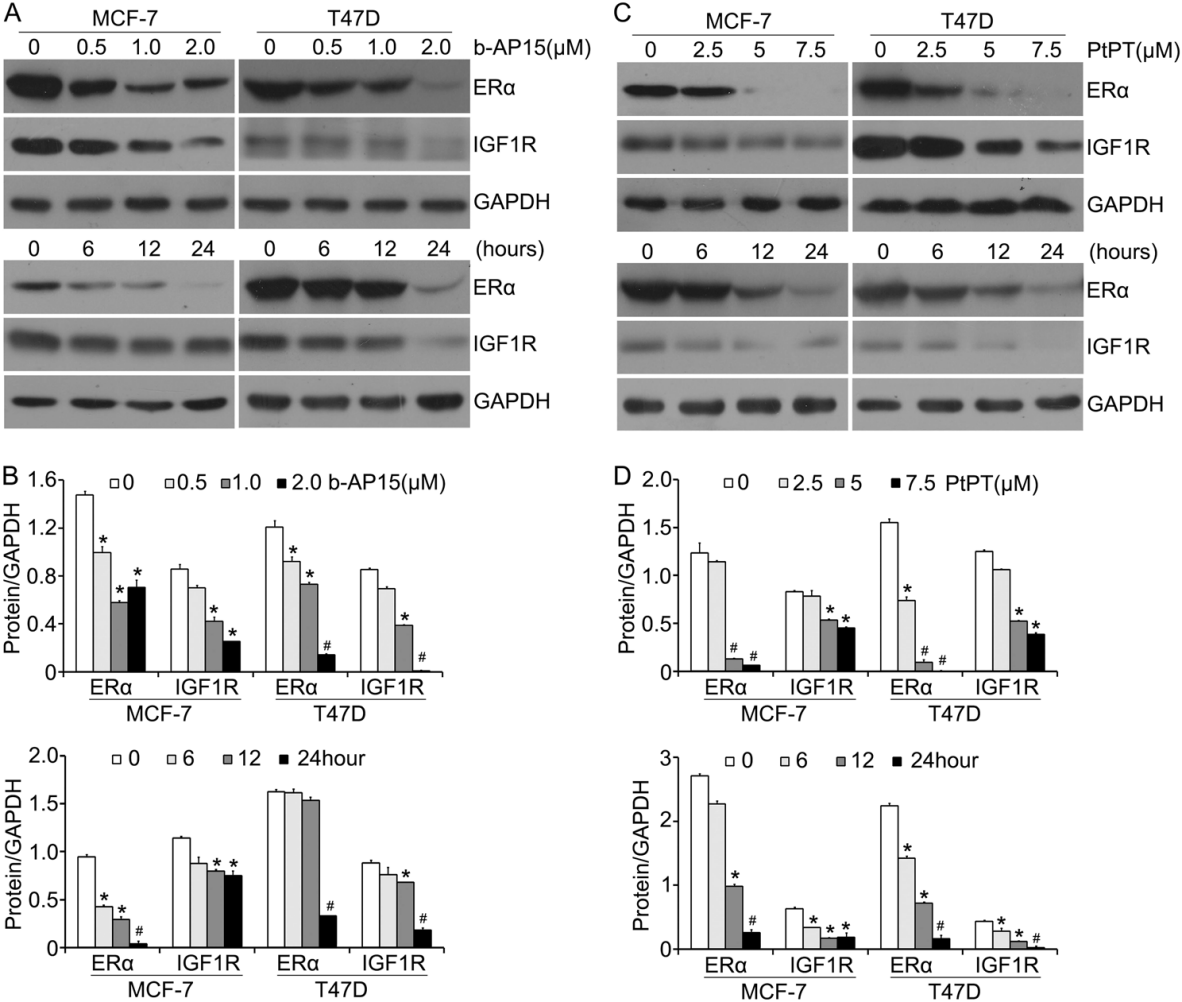
Two inhibitors of USP14 and UCHL5 decrease ERα and IGF1R levels. **a**, **c** BCa cells were exposed to b-AP15 or PtPT for 24 h, or b-AP15 (1 μM) / PtPT (5 μM) for multiple length of time. ERα and IGF1R levels were detected using western blot analysis. The shown images is from one of three independent experiments. GAPDH was probed as a loading control. **b**, **d** Relative quantifications of ERα and IGF1R expression were shown. *p < 0.05, ^#^*p* < 0.01

ERα and insulin-like growth factor type 1 receptor (IGF-1R) signaling pathways are critical in cell proliferation of BCa^17,18^. ERα was validated as the hallmark of anti-endocrine therapy. We next tested whether the two inhibitors of 19 s proteasomes modulate these pathways in BCa cells. Using western blot analysis, we showed that b-AP15 and PtPT dose- and time-dependently downregulated the protein levels of ERα and IGF-1R (Fig. 5). It has been reported that receptor tyrosine kinase(RTKs) involves in cancer progression and development. Given that IGF-1R expression was decreased by b-AP15 and PtPT treatment in BCa cells. To further explore the impact of b-AP15 and PtPT on other RTKs expression, in particular ErbB family members, we detected the expression of EGFR and HER3 proteins. The western blot analysis showed that USP14 and UCHL5 inhibitor decreased the protein levels of EGFR and HER3 (Supplementary Fig. S3a–d). To investigate whether the downregulation of ERα was due to the inhibition of USP14 and UCHL5 in combination, we observed that IU1, an inhibitor of USP14 suppressed a little ERα expression in protein (Supplementary Fig. S4a–c). To further explore the individual role of USP14 in BCa cells, We detected the effect of IU1 on the cell viability using MTS assay. The results showed that inhibition of USP14 has no obvious influence on the cell viability for 48 h (Supplementary Fig. S4d).

**Fig. 6:**
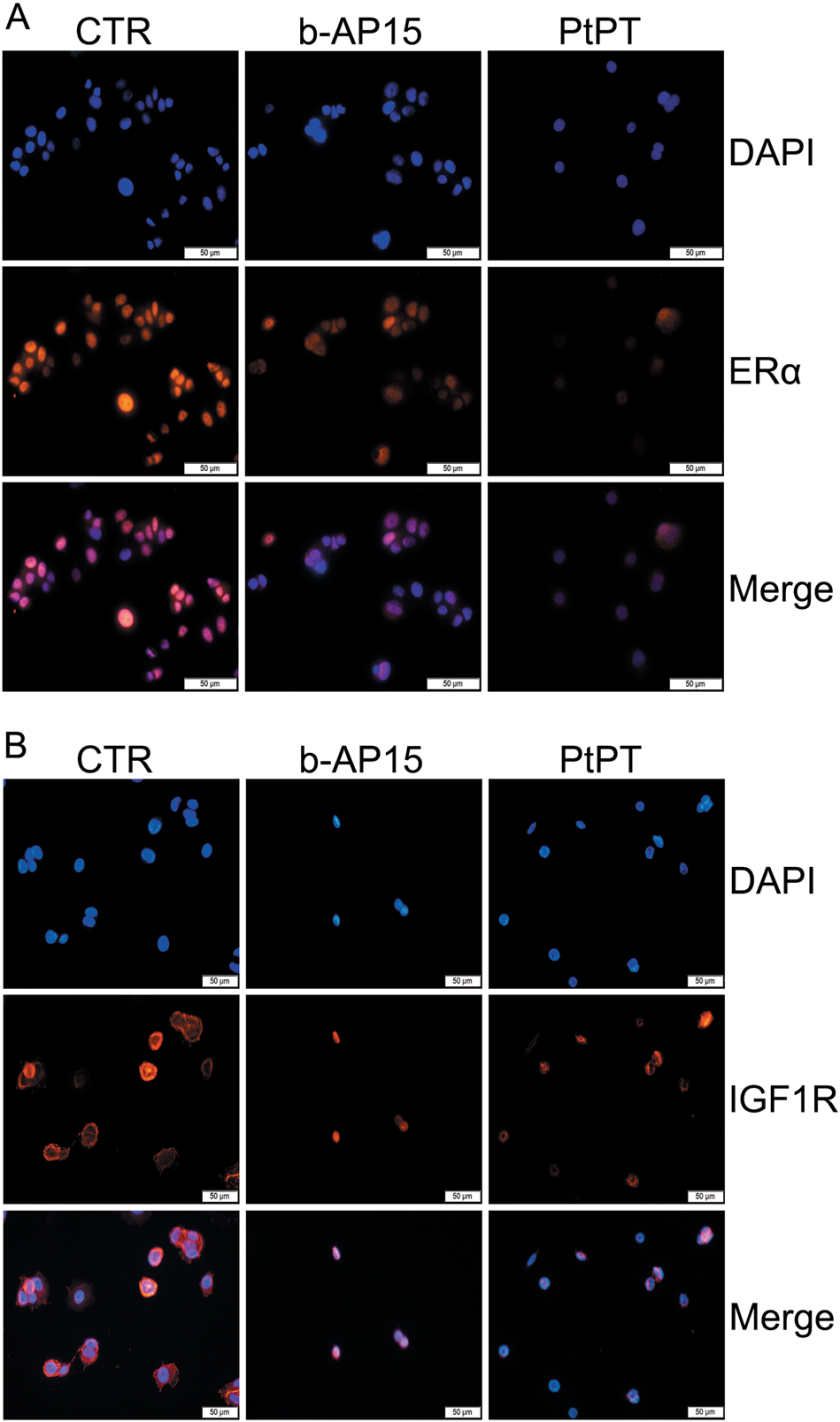
USP14 and UCHL5 inhibitors affect ERα and IGF1R protein levels but not their localization. **a**, **b** BCa cells (**a**: MCF-7; **b**: T47D) post b-AP15 (1 μM) or PtPT (5 μM) treatment for 12 h were fixed and incubated with ERα antibody or IGF1R antibody overnight at 4 °C. Then cells were washed and incubated with secondary Cy3-conjugated antibodies. Nucleus was stained by DAPI. Immunofluorescence microscopy shows endogenous ER α and IGF1R (orange) and nucleus (blue). Three independent experiments were performed

To better understand the distribution of ERα and IGF-1R in ER^+^ BCa cells and to explore whether b-AP15 and PtPT affected their sub-cellular localization, fluorescence microscopy was used to observe protein expression and localization in MCF-7 cells exposed to b-AP15 and PtPT for 12 h. We found that ERα and IGF-1R located in both the nucleus and cytoplasm; however, ERα located mainly in the nucleus. The immunofluorescent staining assay showed that b-AP15 and PtPT decreased ERα and IGF-1R expression in both the nucleus and cytoplasm to a similar extent between the two compartments (Fig. 6).The results demonstrate that b-AP15 and PtPT downregulates total ERα and IGF-1R protein levels.

### USP14 and UCHL5 inhibitors promote ERα degradation and inhibit ERα transcription activity

**Fig. 7:**
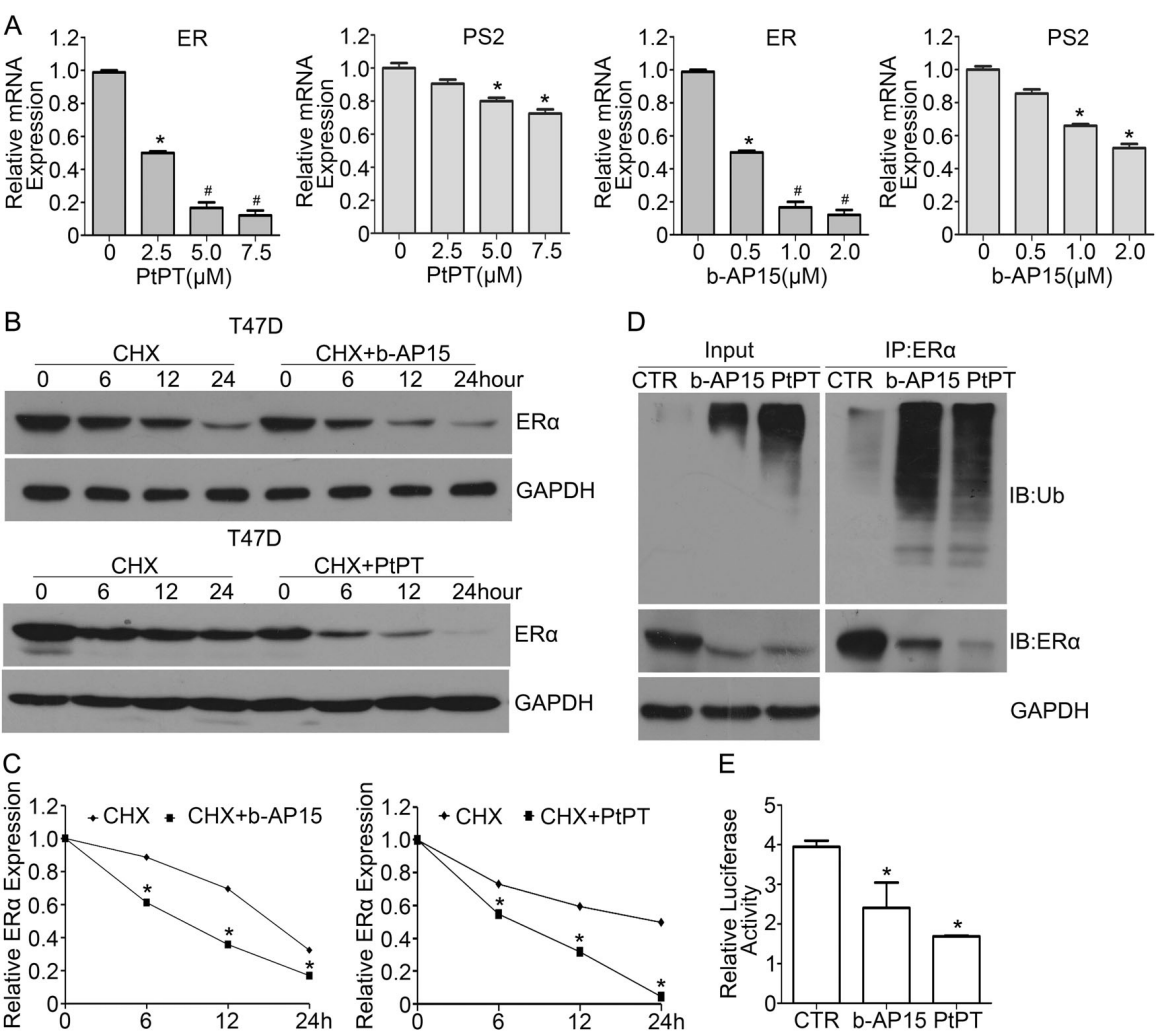
USP14 and UCHL5 inhibitors increase the ubiquitination of ERα and decrease ERα-mediated transcription activity. **a** Total RNAs were collected from T47D cells exposed to b-AP15 or PtPT for 12 h and subjected to RT-qPCR analysis for ERα and ERα-targeted gene, PS2 mRNA expression. Mean ± SD (*n* = 3). *p < 0.05, ^#^*p* < 0.01. **b** T47D cells were treated with cycloheximide (CHX) for 0, 6, 12, 24 h and co-treatment of CHX and b-AP15 or PtPT at the same time. Protein lysates were collected and subjected to western blot assay for ERα protein level. Representative images were shown. **c** Relative quantifications of ERα band density were calculated. *p < 0.05. **d** The total proteins were collected from T47D cells treated with b-AP15 (1 μM) or PtPT (5 μM) for 24 h and MG132 (10 μM) for 6 h before it was collected, immunoprecipitated with ERα antibody beads and immunoblotted for ERα and ubiquitin (Ub). **e** T47D cells were transfected with luciferase reporter plasmid containing estrogen receptor elements (EREs). After transfection for 24 h, cells were exposed to b-AP15 and PtPT for 24 h. Protein lysates were collected and dual-luciferase assay was used to measure relative luciferase activity. Three independent experiments were performed.*p < 0.05

To further explore the mechanisms by which b-AP15 and PtPT downregulates the expression of ERα. We firstly tested whether the two inhibitors have effect on the mRNA expression of ERα. Our PCR results indicated that both b-AP15 and PtPT dramatically decreased ERα mRNA and ERα-targeted gene, PS2 mRNA expression (Fig. 7a), suggesting that b-AP15 and PtPT decrease the transcription of ERα. Besides, we also tested whether ERα protein downregulation by two 19 S inhibitors b-AP15 and PtPT involves enhancing the degradation of ERα using the cycloheximide (CHX) chase assay. The CHX chase assay revealed that in T47D cells the half-life of the ERα is 12 h. Importantly, b-AP15 and PtPT treatment expedited the progressive decreases of ERα protein levels in CHX-treated cells (Fig. 7b, c), indicating that b-AP15 and PtPT promote protein degradation of ERα. To further confirm that b-AP15 and PtPT-induced ERα degradation is through ubiquitin proteasome system, co-IP was used to detect the abundance of ubiquitinated ERα. We found that inhibitors of USP14 and UCHL5 dramatically increased the ubiquitinated ERα (Fig. 7d). In addition, we tested whether b-AP15 and PtPT alter ERα-mediated transcription activity using a luciferase reporter assay. The results reveal that ERα-mediated transcription activity is suppressed by b-AP15 and PtPT (Fig. 7e). Collectively, we suggest that inhibition of USP14 and UCHL5 potently blocks ERα signaling via downregulating the expression and transcriptional activity of ERα.

## Discussion

Increasingly more and more studies showed that DUBs are over-expressed and play critical roles in many cancer cells. Some inhibitors of DUBs have been discovered, such as P5091, a potent and specific inhibitor of USP7 that promotes the degradation of MDM2 and thereby stabilizes p53^19–21^. Recent evidence suggests that deubiquitinases associated with the 19 S proteasome are novel targets for cancer therapy^22^. b-AP15 and PtPT, as inhibitors of USP14 and UCHL5, have been demonstrated that they can induce apoptosis in myeloma, triple negative breast cancer (TNBC) cell lines^23^ and prostate cancer cells^14^. Nevertheless, the effect of these two inhibitors of USP14 and UCHL5 in ERα^+^ BCa cells was not undefined.

BCa is common worldwide, especially in the United States. Sex hormone estradiol participates in breast cancer pathogenesis and most breast cancers are ERα-positive. ERα has become an important target for endocrine therapy in the clinic^24^. Our data showed that b-AP15 and PtPT decreased the expression of ERα at both mRNA and protein levels. Additionally, b-AP15 and PtPT impaired the transcriptional activity of ERα, which represents its prominent function. The ERα signaling pathway stimulates a panel of genes that are associated with poor prognosis and metastasis in ERα^+^ BCa patients. Hence, b-AP15 and PtPT have great potential for further clinical research in BCa therapy.

The current study suggests that two inhibitors of USP14 and UCHL5, b-AP15 and PtPT, dose- and time- dependently suppress cell growth of ERα^+^ BCa cells. In addition, b-AP15 and PtPT induce the activation of caspase 3 which is involved in apoptosis. The family of cysteine proteases was closely related to apoptosis by extrinsic and intrinsic pathways^25,26^. Activation of caspase-3 and PARP cleavage were induced by b-AP15 or PtPT treatment. The Bcl-2 protein family modulates the control of apoptosis in physiology, which makes it a critical target for recent cancer therapy^27,28^. We found that b-AP15 and PtPT downregulated the protein level of anti-apoptotic protein Bcl-2. All of the above evidence indicates that apoptosis induction by b-AP15 and PtPT in BCa cells involves activation of caspase and downregulation of Bcl-2 protein. Subsequently, we further explored the relationship between ER stress and b-AP15 or PtPT-induced cell apoptosis. The ER, as an organelle, mediates proper protein folding, which is essential to the cellular and intercellular communication and metabolism^29^. The accumulation of high levels of misfolded proteins in the ER result in ER stress, which ultimately induces apoptosis of cancer cells. In the current study, b-AP15 and PtPT led to inhibition of the proteasome and triggered ER stress. HSP70 belongs to the heat shock proteins (HSPs), a large family of chaperones that contributes to cell homeostasis and cytoprotection^30,31^. Chaperones play a significant role in cell proliferation, differentiation and cancer development and progression. Due to the formation of more misfolded proteins in cancer cells, HSP chaperonage is more required than normal cells. The expression of phosphorylated eIF2α was increased through combination with GRP78/Bip and PERK under abnormal conditions. Subsequently, the expression of pro-death transcription factor CHOP was upregulated by ATF4^32–35^, which can regulate transcription of pro-survival genes.

The current study has also demonstrated that b-AP15 and PtPT-induced inhibition of cell proliferation was also linked to cell cycle arrest. CDK4 and cyclin D1, which promote G1- to S-phase progression, were downregulated after treatment of b-AP15 and PtPT. Meanwhile, the expression of p21, which restrains the G1- to S- phase transition resulting from hindering the function of cyclin-dependent kinases (CDKs)^36^, was increased by b-AP15 and PtPT treatment. CDKs, as one hallmark of cancer development, play a critical role in fundamental cellular processes, including cell division and gene transcription. It has been reported that CDK4 overexpression has been linked to poor prognosis^37,38^.

More importantly, the expression of anti-apoptotic IGF-1R was decreased after treatment of b-AP15 and PtPT. IGF-1R plays a major role in promoting cell proliferation. Previously studies discovered that inhibition of IGF-1R protein levels is a prime element for suppressing prostate cancer progression via inhibition of angiogenesis and metastasis in the TRAMP mouse model^39^. Additionally, IGF-1R regulates epidermal growth factor receptor (EGFR) and supports cell proliferation in tamoxifen-resistant breast cancer^40^.

The most intriguing discovery is that inhibitors of USP14 and UCHL5 promote the downregulation of ERα protein levels, including its target gene cyclin D1 introduced before. In addition, ERα mRNA and its target gene PS2- the main gene and prognostic indicator in ER-positive breast cancer tissues^41^, were also decreased. Hence, we deduce that b-AP15 and PtPT-induced cell growth arrest and apoptosis are related to inhibition of ERα. Studies have revealed that selective estrogen receptor down-regulators (SERDs), such as fulvestrant, and transcription factor aryl-hydrocarbon receptor (AhR)-mediated ERα suppression is through the ubiquitination/proteasome pathway^42–45^. In this study, we found that b-AP15 and PtPT increase ubiquitinated ERα. It is known that b-AP15 and PtPT can inhibit the activity of both USP14 and UCHL5 simultaneously, implying that the downregulation of ERα by b-AP15 and PtPT may attribute to the suppression of USP14 and UCHL5. Here, we suggest that b-AP15 and PtPT downregulate ERα via promoting its degradation and inhibiting its transcription. ERα is a major prognostic marker and a prominent target for pharmacological therapy in ERα^+^ BCa. Therefore, the effect of PtPT and b-AP15 targeting ERα should be valued in BCa and will provide novel agents in ERα regulation.

Currently, patients undergo Fulvestrant and tamoxifen treatment in clinical have gain acquired resistance, even irresponsive to these therapies. A published phase II study reported that proteasome inhibitor bortezomib makes a contribution to a clinical benefit rate of 22% in progressive metastatic- and endocrine-resistant BCa, suggesting that 20 S proteasome inhibition provides a critical therapeutic strategy for breast cancer patients^46^. In the current study, we suggest that 19 S proteasome inhibitors b-AP15 and PtPT can induce growth arrest and apoptosis in ER^+^ BCa cells, and have the tremendous potential for endocrine therapies-resistant ERα^+^ breast cancer or early stage ERα^+^ BCa.

## Materials and methods

### Materials

b-AP15, bortezomib and IU1 were purchased from Selleckchem (Houston, TX, USA). PtPT was synthesized as we previously described^13^. The 17β-estradiol (E2) and tamoxifen were obtained from Sigma (St. Louis, MO. USA).Antibodies was as follows: anti-ERα (8644 s),anti-PARP (9542 s),anti-Bcl-2 (15071 s), anti-caspase cleave 3 (9661 s), anti-CDK4 (1279 s), anti-cyclinD1 (29269), anti-p21 (2947p), anti-CHOP (2895 s), anti-eIF2α (53249D7D3), phospho-eIF2α (3398Sd9g8), anti-HSP70 (4873), anti-ubiquitin (3936 s), anti-K48-linkage Specific Polyubiquitin (8081 s), anti-IGF1R anti-HER3 (12708T) and anti-EGFR (4267 S) were from Cell Signaling Technology (MA,USA). Anti-GAPDH was from Bioworld Technology (St.Louis Park,MN,USA). MTS assay (CellTiter 96 Aqueous One Solution reagent) and Dual-Luciferase Reporter Assay was purchased from Promega Corporation (Madison,WI,USA). Propidium iodide (PI) and Annexin V-FITC Apoptosis Detection Kit were purchased from Keygen Company (Nanjing,China). Dynabeads antibody coupling kit was purchased from Life technologies. ER-luciferase reporter plasmid was from Yesen Company (Shanghai,China).

### Cell lines and culture conditions

Human breast cell lines MCF-7 and T47D were obtained from ATCC (Manassaa,VA,USA). MCF-7 cells were cultured onto 75 cm^2^ cell culture flask in Hyclone DMEM (Thermo Fisher Scientific) supplemented with 10% FBS (Biological Industries) and 10 mg/ml insulin at 37 °C and in a humidified atmosphere of 5% CO_2_. T47D cells were cultured as MCF-7 cells without insulin.

### Cell viability assay

This assay was performed according to previous description^47^. When cells are in exponential growth, MCF-7 and T47D cells were plated onto the 96-well plates at 2 × 10^4^ cells/ml. After incubation for 24 h, cells were treated with b-AP15 and PtPT for 24 h, 48 h or 72 h. A concentration of 20 μl MTS was added into plates and cultured for 3 h in dark. The absorbance of optical density was measured with a microplate reader (Sunrise, Tecan, Mannedorf, Switzerland).

### Colony formation assay

MCF-7 and T47D cells were treated with increasing concentrations of b-AP15 and PtPT for 24 h, then cells were suspended in 30% agarose supplemented with 20% FCS and 50% Hyclone DMEM in 60 mm dishes and cultured in atmosphere of 5% CO_2_. After 10–14 days, cells were stained with 0.3% crystal violet solution. The colonies > 60 μm were counted under a light microscope. The experiments were performed in three times.

### Flow cytometry analysis of Cell cycle and apoptosis

For cell cycle assay, MCF-7 and T47D cells were treated with b-AP15 and PtPT for 0 h, 6 h, 12 h and 24 h. Cells were digested and collected under centrifugation, discarding supernatant. Cells were then washed with cold PBS thrice. Precipitated cells were resuspended with PBS and 70% ethanol at a ratio of 1:4. Cells were fixed at 4 °C overnight and washed with cold PBS twice again, followed by incubation with PI, RNase A and 0.2% Triton X-100 complexes for half an hour at 4 °C in dark.

Apoptosis was detected using Annexin V-fluoroisothio-cyanate (FITC)/PI staining as previous description^48^. Breast cancer cells were treated with b-AP15 and PtPT for 24 h. Cells were digested with pancreatic enzymes and centrifuged for 5 min.The collected cells were washed with 4 °C PBS twice and the precipitated cells were resuspended with 500ul binding buffer, followed by Annexin V-FITC incubation for 15 min and PI staining for another 15 min in dark. Stained cells were analyzed through flow cytometry analysis.

### Western blot analysis

This assay was performed as we previously reported^49^, protein lysates from breast cancer cells treated with b-AP15 and PtPT were extracted in RIPA lysis buffer (Thermo Scientific) supplemented with protease and phosphatase inhibitor (Nanjing, China) and PMSF. Collected proteins were quantitated and then separated by SDS-PAGE. Proteins in PAGE were then transferred to PVDF membranes. 5% defatted milk powder in phosphate buffer saline was used to blocked blots for 1 h. The membranes were then washed with PBS-T and incubated with primary antibodies. After incubation overnight, membranes were washed with PBST thrice for 6 min and incubated with secondary antibodies for 1 h. The membranes were washed as before and exposed to X-ray films (Kodak, Japan) by the use of ECL detection reagents.

### Co-immunoprecipitation

The kit was used to examine protein interactions as we reported previously^50^. Dynabeads coupled with antibodies (1:50) for 16–24 h and then quantitative cell lysates were added for addition 1 h with end-to-end rotation at 4 °C. The immunocomplexes were washed with PBST thrice. The dynabeads adsorbed by magnetic traps were dissolved with SDS blue loading buffer. Bound proteins were eluted by incubation for 10 min at 70 °C, and then subjected to SDS-PAGE and western blot analyses.

### Immunofluorescence assay

To observe the subcellular localization and expression of objective protein, cells were seeded on chamber slide and treated with b-AP15 and PtPT for 24 h. The cells were washed with 4 °C PBS and fixed with 4% paraformaldehyde for 10 min, followed by permeabilization with 0.1% Triton X-100 (Solarbio Life Science) diluted with PBS for 5–10 min. Then cells were blocked with 5% BSA for 30 min and incubated with primary antibody diluted with 1% BSA overnight at 4 °C, followed by incubation of secondary Cy3-conjugated antibody and fluoroshield mounting medium with DAPI (Abcam).Image were captured using fluorescence microscope.

### PCR analysis

To evaluate mRNA levels of ERα and PS2, RNAs were isolated from T47D cells as we previously described^50^. The concentration and purity of RNAs were measured at 260:280 nm. After then, the concentration of RNAs were diluted as same. The first-strand cDNA was synthesized with 1 μg total RNA. The mRNA expression levels of GAPDH, ERα and PS2 were measured by Real-time PCR with SYBR Premix Ex TaqTM kit (TaKaRa, Dalian, China).PCR primer are as following:^51^ ER: F: 5′-TCTTGGACAGGAACCAGGGA-3′; R: 5′-CAGAGACTTCAGGGTGCTGG-3′; PS2: F: 5′-TTGTGGTTTTCCTGGTGTCA-3′; R: 5′-GCAGATCCCTGCAGAAGTGT-3′; GAPDH: F: 5′-TCCCATCACCATCT TCCA-3′; R: 5′-CATCACGCCACAGTTTCC-3′.

### Luciferase reporter assay

Estrogen receptor luciferase reporter plasmids were introduced to BCa cells with Lipofectamine 2000 (Invitrogen). The RPMI opti-MEM was replaced with 10% FBS DMEM medium after 6–8 h, then cells were treated with DMSO,b-AP15 and PtPT at 24 h post-transfection for 24 h. Passive Lysis Buffer, Luciferase Assay Buffer II and Stop & Glo Reagent were prepared and the activity of luciferase was measured according to instruction. The relative luciferase was calculated by firefly luciferase to Renilla luciferase (Mean ± SD, *n* = 3).

### Statistical Analysis

The data are presented as Mean ± SD from three independent experiments where applicable. Unpaired Student’s t-test or one way ANOVA is used where appropriate. Statistical analysis was performed by SPSS 16.0. P < 0.05 was considered statistically significant.

## Electronic supplementary material

Supplementary Figure legends
