# Supplementary material for: Targeting proteasome-associated deubiquitinases as a novel strategy for the treatment of estrogen receptor-positive breast cancer
Source: Oncogenesis. 2018 Sep 24;7(9):75. doi: 10.1038/s41389-018-0086-y (PMC6155249; doi:10.1038/s41389-018-0086-y)
Supplement: Supplementary file 1 — Supplementary Figure legends [file 41389_2018_86_MOESM1_ESM.docx]

**Supplementary materials：**

**
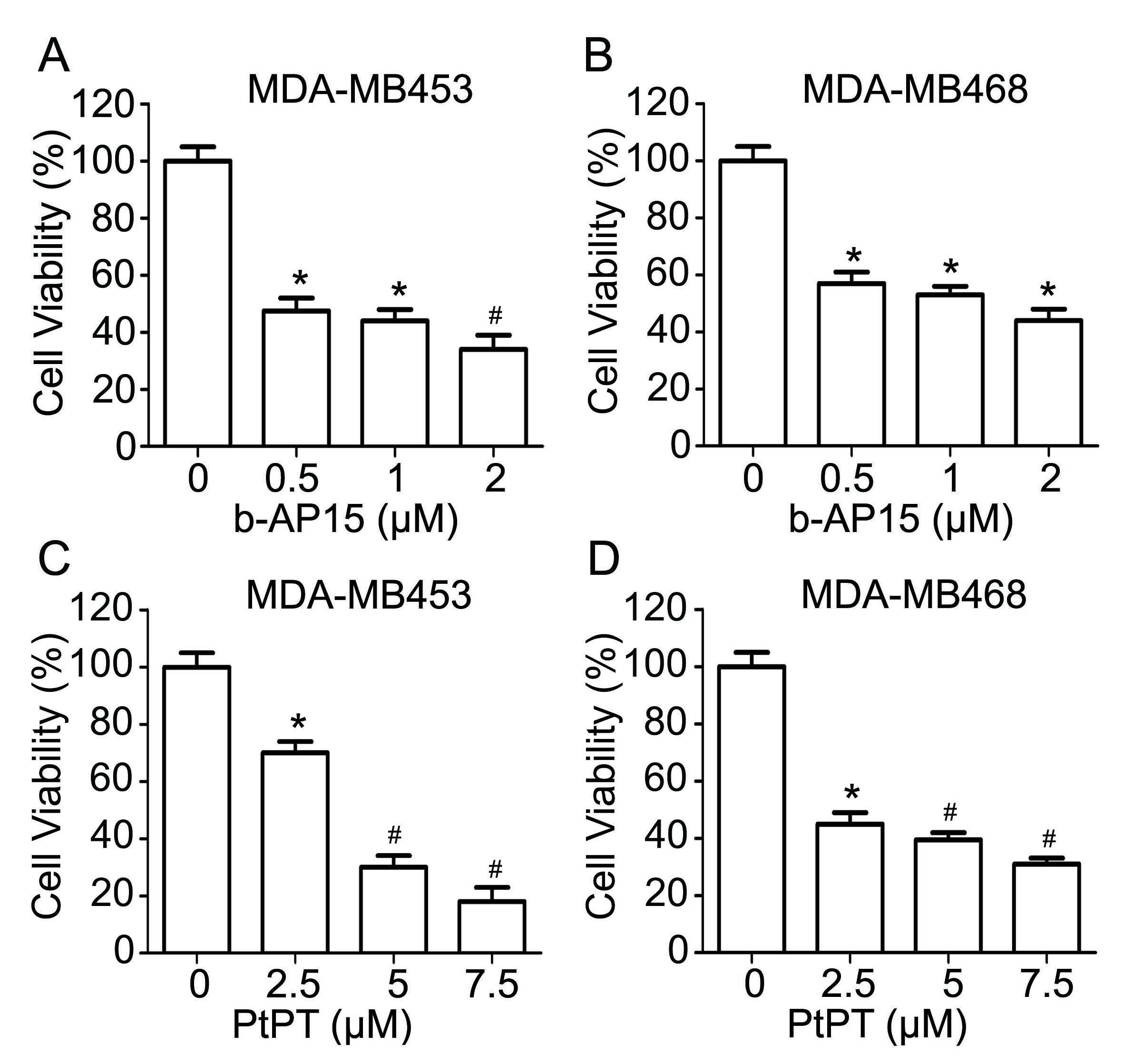
**

**Fig. S1 USP14 and UCHL5 inhibitors suppress the growth of TNBC cells. a**, **b** MDA-MB453 and MDA-MB468 cells were treated with b-AP15 for 48h, cells viability was determined using MTS assay. **c**, **d** MDA-MB453 and MDA-MB468 cells were exposed to PtPT for 48h, cell viability was determined using MTS assay. Mean ± SD of three independent experiments. *p<0.05, ^#^p<0.01, the two-sided t-test.


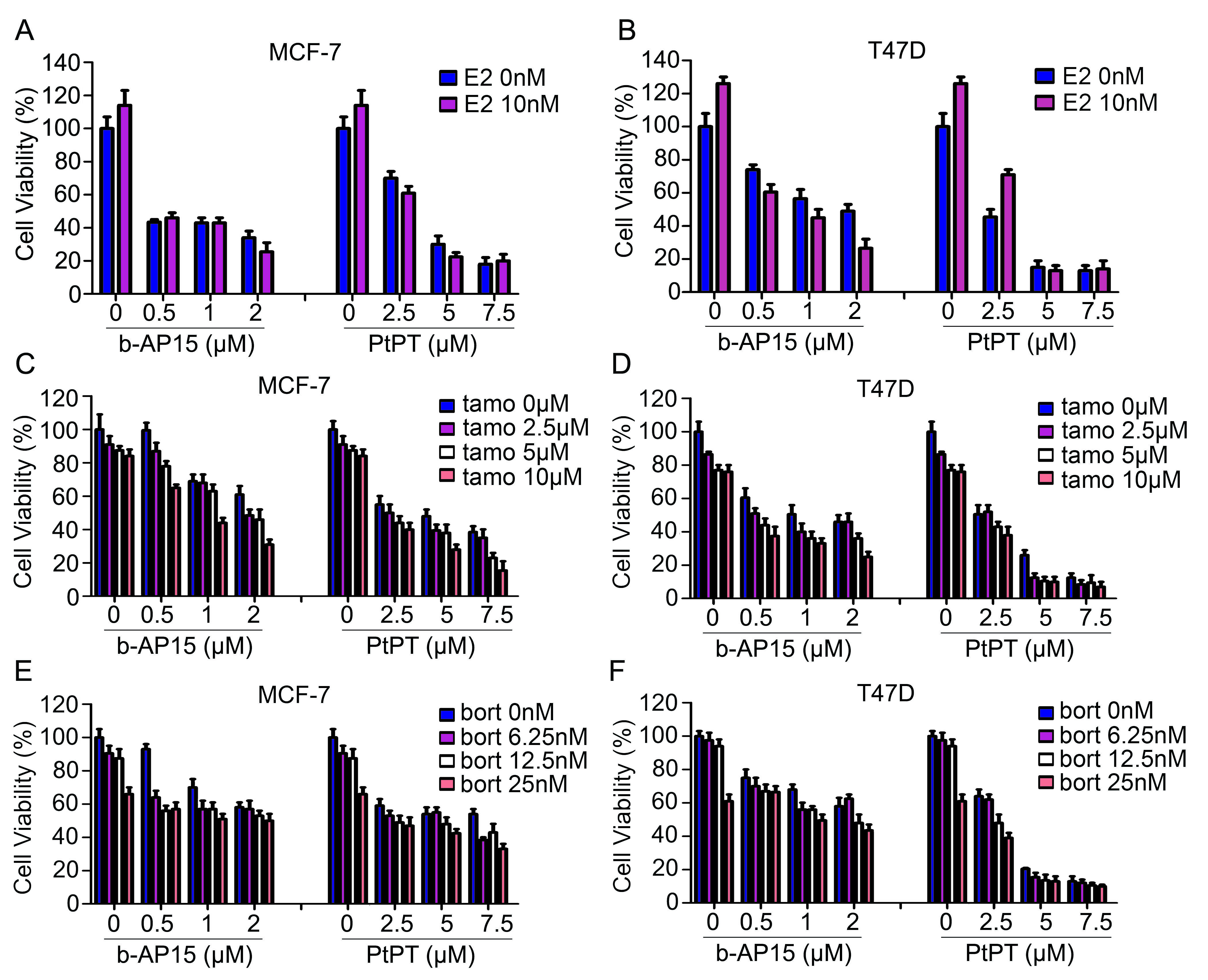


**Fig. S2 Synergistic effect of ER^+^ BCa cells by combination of b-AP15 or PtPT and other** **small molecular compounds**. **a**, **b** MCF-7 and T47D cells were seeded into 96 wells plates. Cells were treated with b-AP15/PtPT in the presence or absence of estrogen at 10nM for 48h. Cell viability was measured using MTS. ERα^+^ BCa cells were exposed to b-AP15/PtPT or **c**, **d** tamoxifen/ **e**, **f** bortezomib alone or to drug-combination for 48h. Cell viability was detected using MTS.


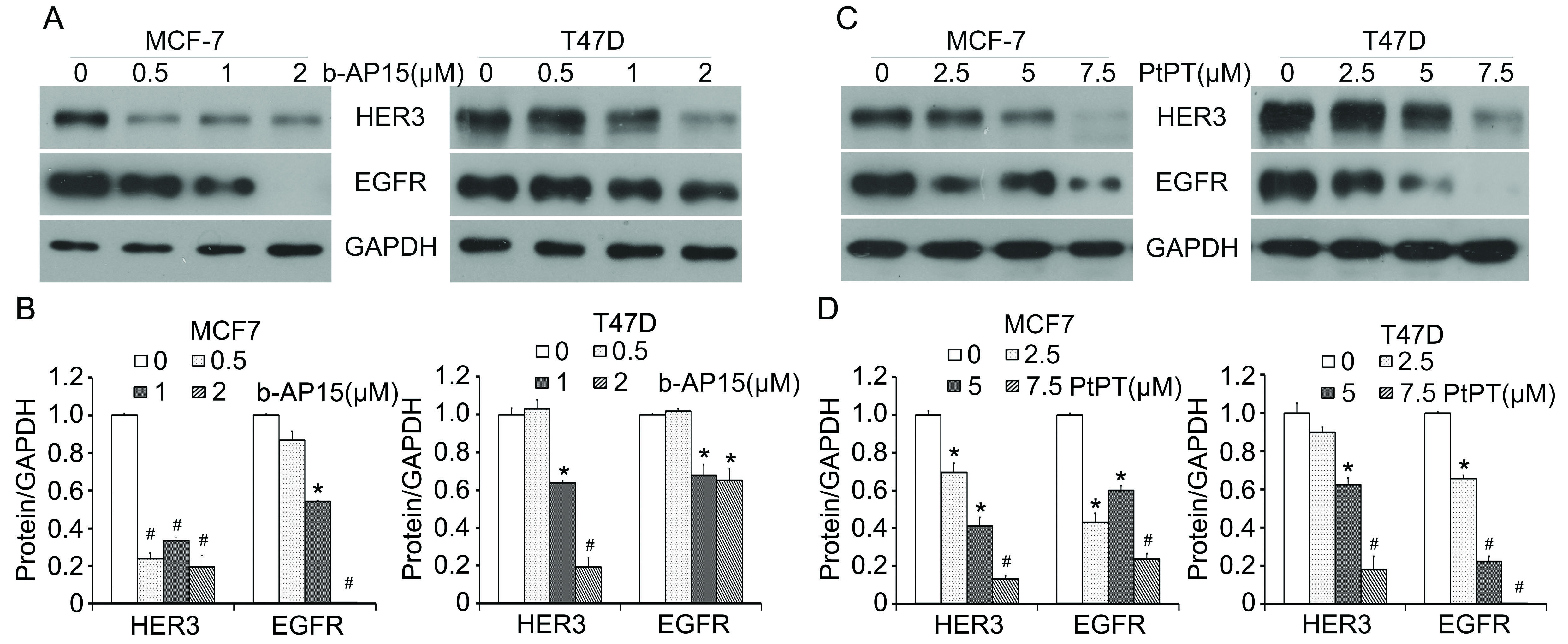


**Fig. S3 USP14 and UCHL5 inhibitors downregulate RTKs expression. a**, **c** BCa cells were treated with b-AP15 and PtPT for 24h. Protein lysates were collected and subjected to western blot analysis for EGFR and HER3. GAPDH was shown as a loading control. **b**, **d** Relative quantifications of EGFR and HER3 expression were shown. *p<0.05, ^#^p<0.01.


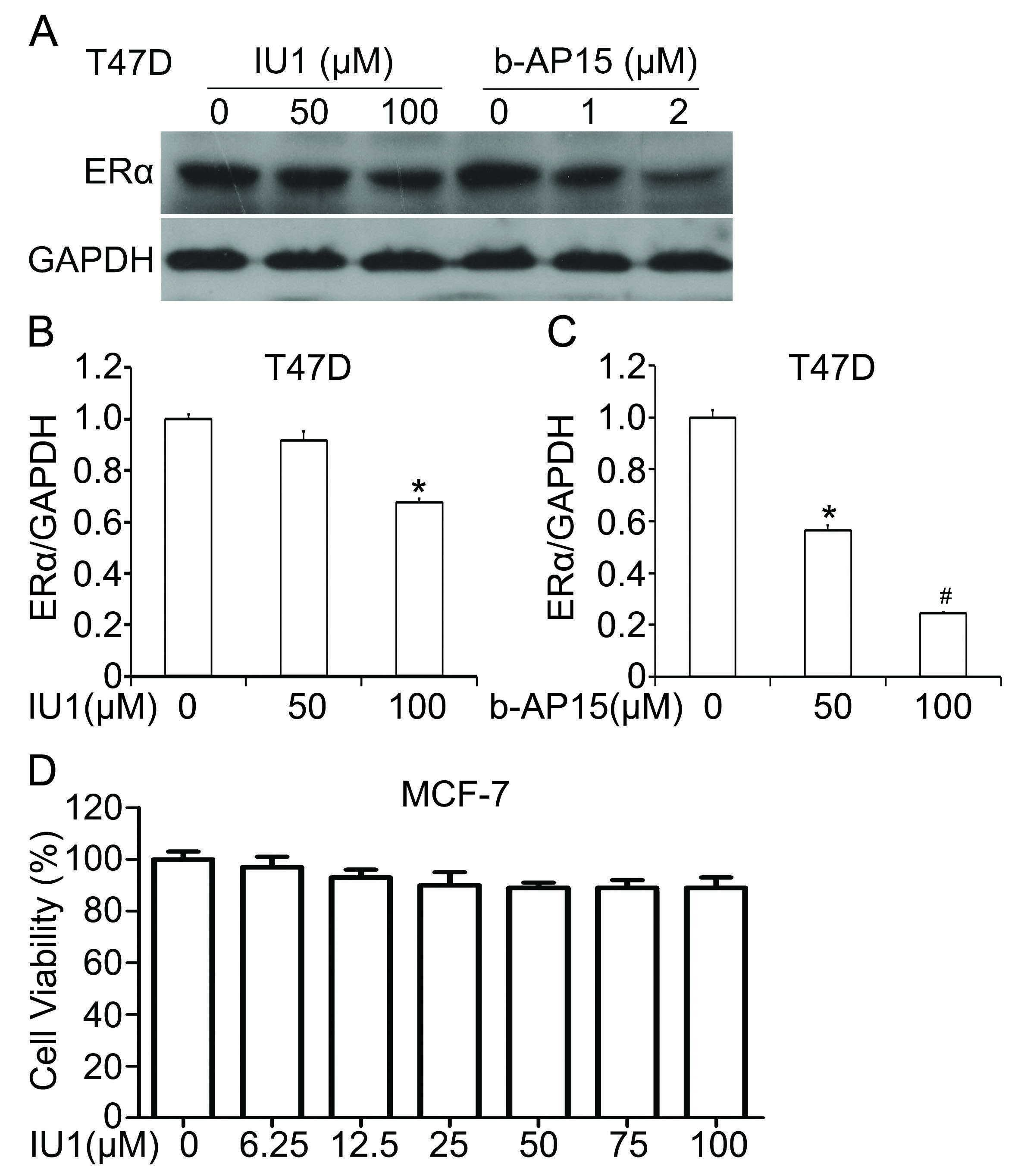


**Fig. S4 The inhibitory effect of USP14 inhibitor alone is slight compared to b-AP15 on the expression of ERα and cell viability. a** T47D cells were treated with IU1 or b-AP15 at various doses for 24h. Western blotting was conducted to detect the expression of ERα. GAPDH was shown as a loading control. **b**, **c** Relative quantifications of ERα band density were calculated. *p<0.05, ^#^p<0.01. **d** MCF-7 cells were exposed to IU1 (6.25, 12.5, 25, 50, 100uM) for 48h. Then cell viability was measured using MTS. Mean ± SD of three independent experiments.
